# Magnetic resonance imaging could precisely define the mean value of tendon thickness in partial rotator cuff tears

**DOI:** 10.1186/s12891-023-06756-5

**Published:** 2023-09-09

**Authors:** Umile Giuseppe Longo, Sergio De Salvatore, Giuliano Zollo, Giovanni Calabrese, Ilaria Piergentili, Mattia Loppini, Vincenzo Denaro

**Affiliations:** 1 Research Unit of Orthopaedic and Trauma Surgery, Fondazione Policlinico Universitario Campus Bio-Medico, Via Alvaro del Portillo, Roma, 200 - 00128 Italy; 2 Research Unit of Orthopaedic and Trauma Surgery, Department of Medicine and Surgery, Università Campus Bio-Medico di Roma, Via Alvaro del Portillo, Roma, 21 - 00128 Italy; 3 Department of Orthopedics, Children’s Hospital Bambino Gesù, Palidoro, Rome, 00165 Italy; 4 IRCCS Humanitas Research Hospital, Rozzano, 20089 Italy; 5 Department of Biomedical Sciences, Humanitas University, Pieve Emanuele, 20072 Italy

**Keywords:** Rotator cuff tear, Orthopaedic surgery, Tendon anatomy, Full-thickness rotator cuff tears, Partial-thickness rotator cuff tears, Rotator cuff tendon, Tendon thickness, Tendon length

## Abstract

**Purpose:**

Rotator Cuff (RC) lesions are classified in full-thickness and partial-thickness tears (PTRCTs). To our knowledge, no studies investigated the mean size of shoulder tendons in healthy and PTRCT patients using MRI scans. The aim of the study was to provide data to obtain and compare the mean value of tendon sizes in healthy and PTRCTs groups.

**Methods:**

From 2014 to 2020, 500 were included in the study. They were divided into two groups: Group 1 (100 subjects) was composed of people positive for partial-thickness rotator cuff tears (PTRCTs), while the 400 subjects in Group 2 were negative for PTRCTs.

**Results:**

Overall, of the patients included in the study, 231 were females and 269 were males. The mean age of the patients was 49 ± 12.7 years. The mean thickness of the supraspinatus tendon (SSP) was 5.7 ± 0.6 mm in Group 1, 5.9 ± 0.6 mm in Group 2 (p < 0.001). The mean length of the ISP tendon was 27.4 ± 3.2 mm in Group 1, 28.3 ± 3.8 mm in Group 2 (p = 0.004). The mean width of the SSP tendon was 17 ± 1.6 mm in Group 1, 17.6 ± 2 mm in Group 2 (p = 0.004). The mean width of the infraspinatus tendon (ISP) tendon was 17.7 ± 1.4 mm in Group 1, 18.3 ± 2.1 mm in Group 2 (p = 0.02).

**Conclusion:**

The anatomical data present in this paper may serve as a tool for surgeons to properly manage PTRCTs. The findings of the present study aimed to set the first step towards reaching unanimity to establish international cut-off values to perform surgery. Additionally, they could widely increase diagnostic accuracy, improving both conservative and surgical approaches. Lastly, further clinical trials using more accurate diagnostic MRI tools are required to better define the anatomical differences between PTRCT and healthy patients.

**Level of evidence:**

Level II, Retrospective Comparative Trial

## Introduction

The financial burden of the Rotator cuff tears (RCT) is relevant in industrialized countries, representing the second most costly problem in the worker’s compensation system [1–3]. It has been estimated that shoulder pain in RCT patients is the cause of 4.5 million consultations and over 250,000 surgeries in the United States [4]. Moreover, shoulder pain is ranked as the third most common musculoskeletal problem [5, 6], causing significant discomfort for the patient with a reduced quality of life [7–9]. According to the amount of tendon tissue involvement, these lesions are classified in full-thickness and partial-thickness rotator cuff tears (PTRCT) [10]. Full-thickness tears encompass the entire cross-sectional area of the tendon tissue [11], possibly producing pain and loss of function in the affected shoulder, as many patients are asymptomatic [12]. While full-thickness lesions frequently require surgical treatment, no consensus has been reached on a single computative method for treating symptomatic PTRCTs [13]. Compared to full-thickness tears, partial-thickness ones are more frequent [14]. Although rotator cuff tear progression can be difficult to predict [15], partial lesions tend to develop into full-thickness ones over few years [16–19]. Moreover, uncertainties arise due to many tears being asymptomatic [20].

Though controversial, there is a consensus to perform a surgical repair of lesions involving more than 50% of the tendon thickness in symptomatic or athletic patients who have failed conservative treatment [21–23]. However, the opinion of the scientific community is not unanimous and the 50% rule has received limited support [24]. Furthermore, the surgical technique of choice is influence by the thickness, size and morphology of the tear [25].

Even though new methods, such as intra-articular depth guide, have shown improved accuracy in measuring the percentage of PTRCTs [26], it is still not possible to compare the size of an RC tear without standard values. These parameters would allow the surgeon to clearly define PTRCTs exceeding 50% of tendon depth. Numerous anatomical studies focused on the insertional footprint of the rotator cuff with the purpose of defining such parameters [27–31]. However, the available studies on this topic have several limitations. Cadaveric studies do not assess the exact thickness of the thin RC tendons, as minor variations of thickness are hardly detected at a gross examination. These studies may also fail in considering intratendinous partial lesions and disclosing the age and sex of each cadaver used. Furthermore, the use of cadaveric specimens is associated with high costs, and the available studies include a small sample size [29], making it difficult to have a significant amount of cases.

Conversely, magnetic resonance imaging (MRI) showed sensitivity and specificity values of 95% in detecting both complete and partial-thickness RC tears [20, 32–35] and also has high accuracy and diagnostic validity for a description of tear size and location [36–38]. However, to our knowledge, no studies investigated the mean size of shoulder tendons in healthy and PTRCT patients. Furthermore, as described by Malavolta et al., MRI displays the lowest detection accuracy for SSC tears among all rotator cuff tendon tears [39].

In this study, the RC tendon’s length, width, and thickness have been measured using MRI. The primary purpose of this study was to use MR imaging to characterize the length, width, and thickness of rotator cuff tendons in healthy patients. Secondarily, we compared these parameters for patients with and without asymptomatic partial thickness rotator cuff tears to evaluate for differences between these groups. The hypothesis was that different measurements would result among the two mean values of tendon sizes in healthy and PTRCTs groups. This study should be addressed as diagnostic rather than clinical, with the aim of contributing to the assessment of a precise tendon measurement range.

## Methods

### Eligibility criteria

From January 2014 to December 2020, 1758 subjects underwent a nuclear magnetic resonance (MRI) of the shoulder in our Institution. Following the application of inclusion and exclusion criteria, 500 patients were enrolled, 100 were positive for PTRCT, and 400 were negative.

Patients were included in this retrospective comparative trial if the following conditions were present at the time of MRI evaluation: age ranging from 20 to 80 years old, no RC tear diagnosed on clinical grounds, no episodes of shoulder instability, no history of biceps or shoulder loss of function.

Patients were excluded if the following conditions were detected during MRI evaluation: RC full-thickness rotator cuff tears, biceps tendon subluxation or complete rupture, inflammatory joint disease, a sign of fracture of the humeral glenoid or grater/lesser tuberosities, surgery on the examined or contralateral shoulder, labral pathology.

Patients were divided according to their pathology into two groups. Group 1 included patients positive for PTRCT, constituted by 51 females and 149 males, with a mean age of 54.2 (± 12) years old. Conversely, group 2 included patients negative for PTRCT, constituted by 180 females and 120 males, with a mean age of 47.4 (± 13) years old.

### MRI evaluation

Patients were positioned supine, with arms at the side of the body and forearms pronated to bring the thumbs to a forward stance. Images were obtained by a 1.5T unit (Siemens Somaton Sensation). All the patients underwent an MRI of the shoulder, which included the following sequences: SE T1-weighted obtained in the coronal plane (TR 456ms, TE 13ms, slice thickness 3 mm); TSE T2-weighted obtained in the coronal plane (TR 2500ms, TE 95ms, slice thickness 3 mm); PD obtained in the coronal plane (TR 2500ms, TE 14ms, slice thickness 3 mm); GRE obtained in the axial plane (TR 650ms, TE 20ms, slice thickness 3 mm) and sagittal plane (TR 700ms, TE 21ms, slice thickness 3 mm).

**Fig. 1:**
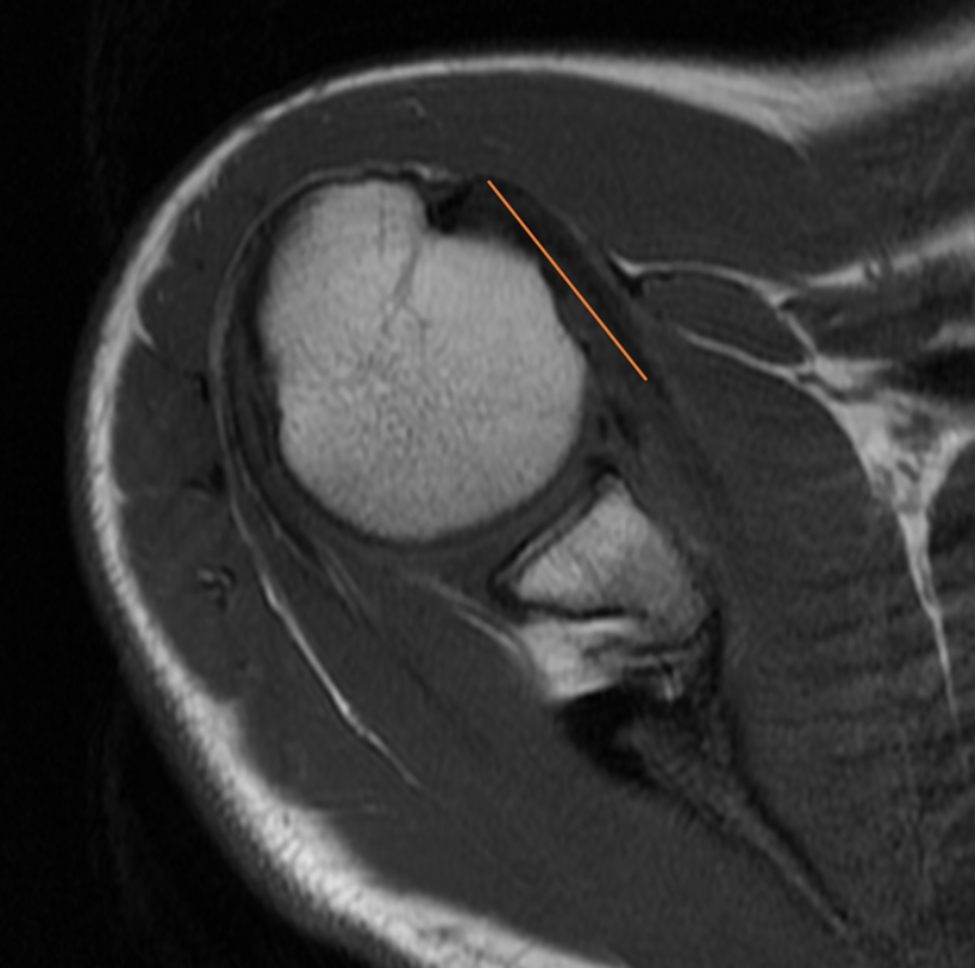
The length of the subscapularis (SSC) tendon was measured on the axial TSE sequence, from the myotendinous junction to its insertion along the medial aspect of the biceps groove

**Fig. 2:**
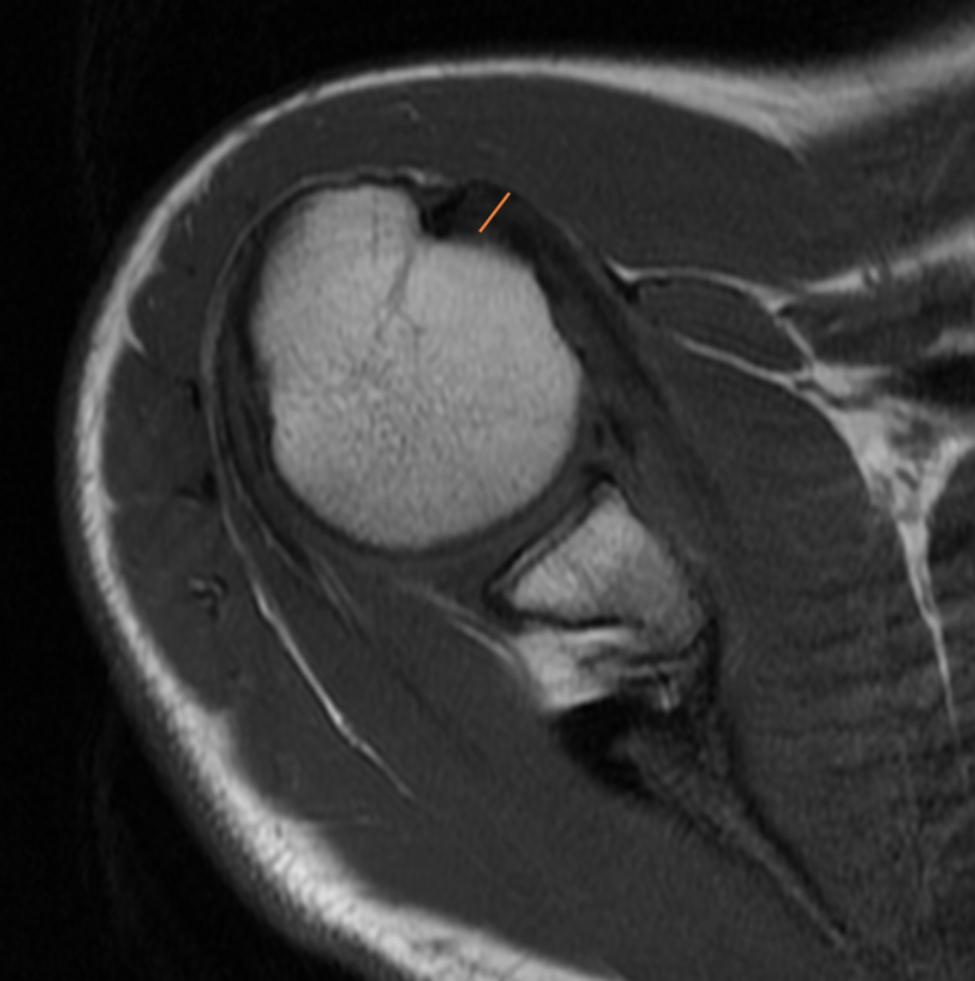
The thickness of the SSC tendon was measured at the insertion of the tendon on the humeral footprint

**Fig. 3:**
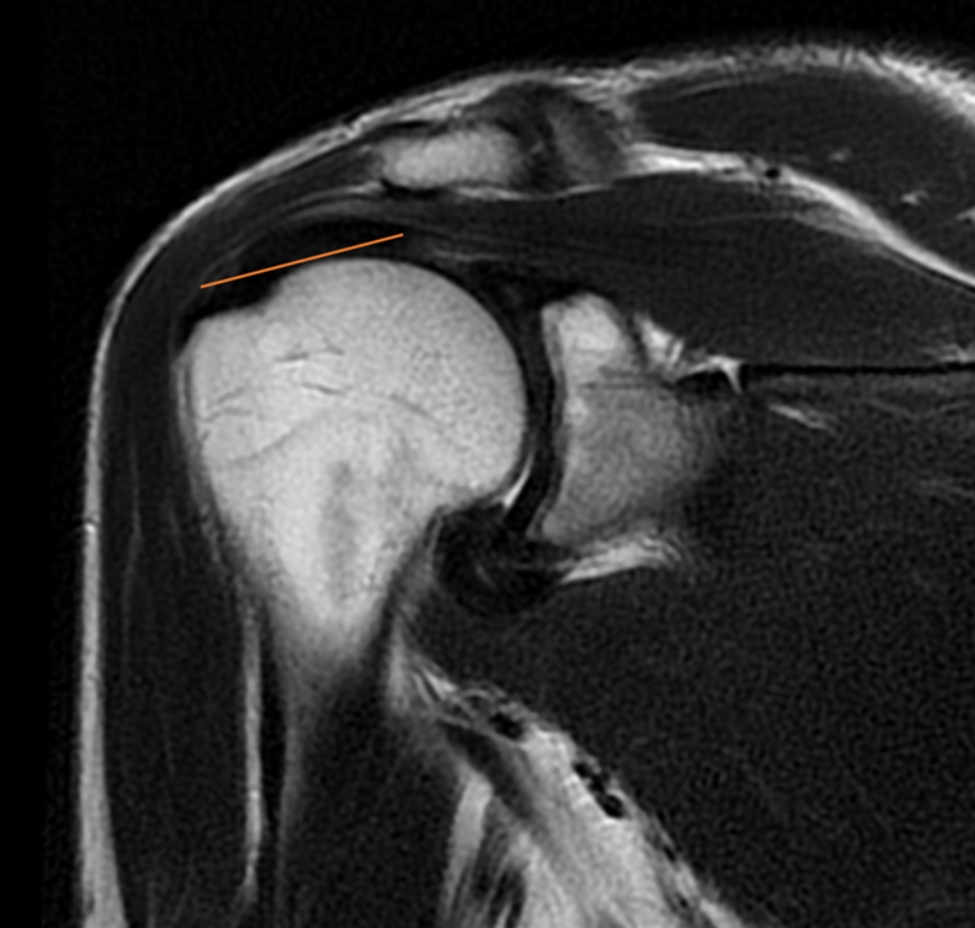
The length of the supraspinatus (SSP) tendon was measured in the coronal plane

**Fig. 4:**
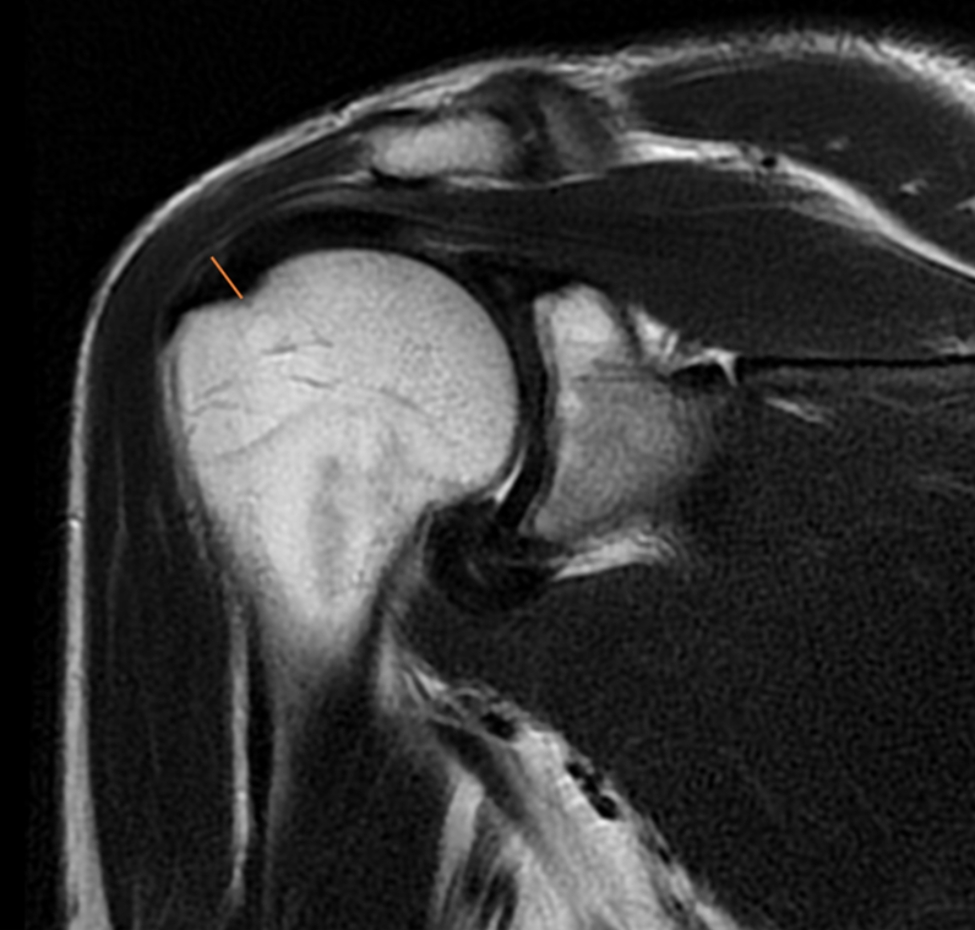
The thickness of the SSP tendon was measured at the insertion of the tendon on the humeral footprint

**Fig. 5:**
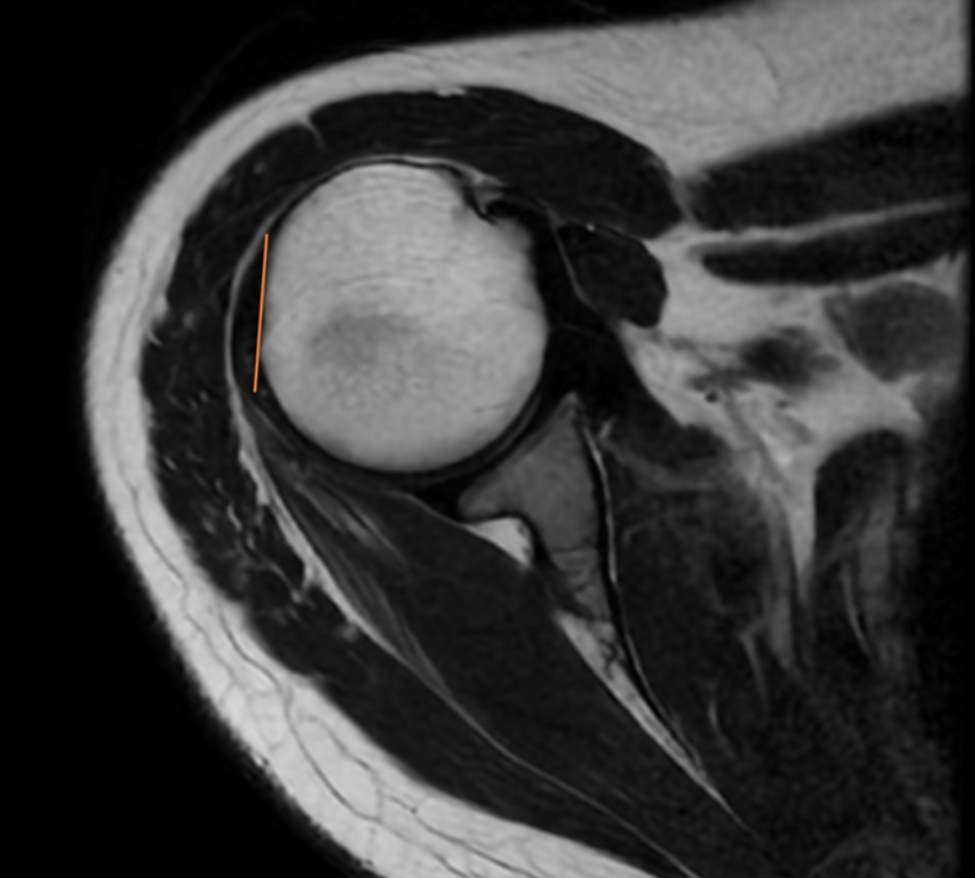
The length of the infraspinatus (ISP) tendon was measured on the axial sequence, from the myotendinous junction to its insertion on the greater tuberosity of the humerus

**Fig. 6:**
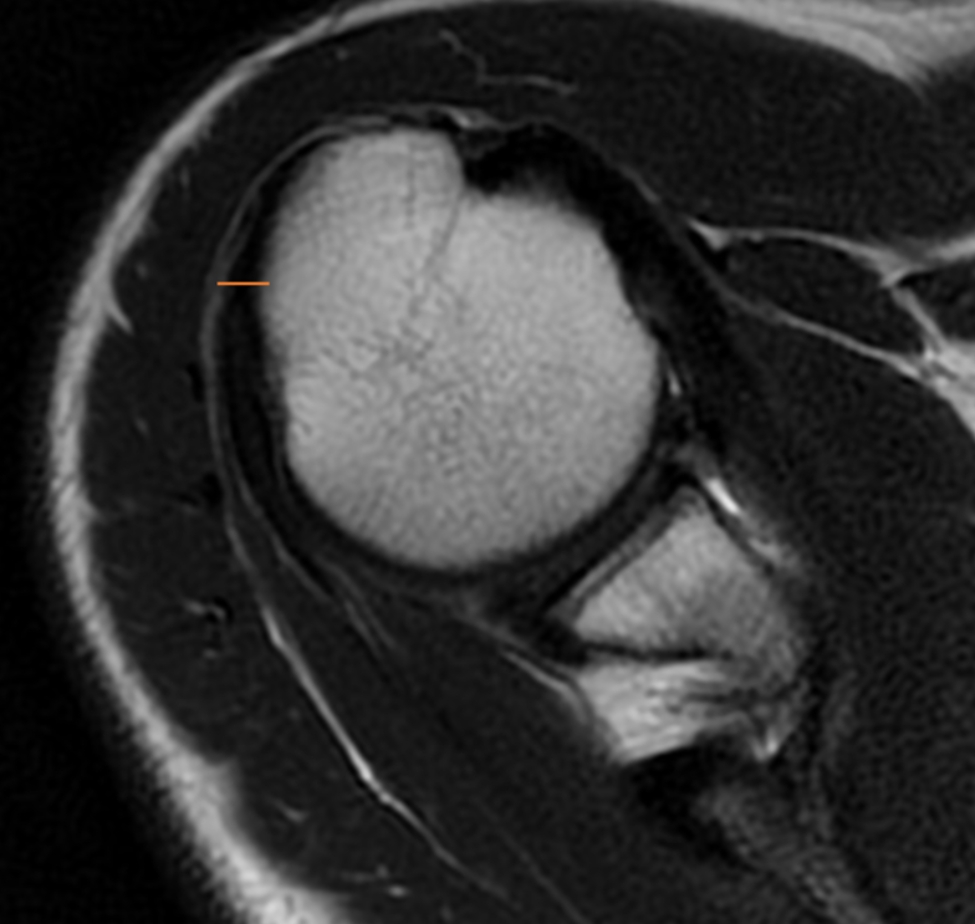
The thickness of the ISP tendon was measured at the insertion of the tendon on the humeral footprint

The length of the subscapularis (SSC) tendon was measured on the axial TSE sequence, from the myotendinous junction to its insertion along the medial aspect of the biceps groove (Fig. 1). The thickness of the SSC tendon was measured at the insertion of the tendon on the humeral footprint (Fig. 2). The supraspinatus (SSP) tendon length was measured using the TSE T2-weighted sequence obtained in the coronal plane (Fig. 3); the thickness of the SSP tendon was measured at the insertion of the tendon on the humeral footprint (Fig. 4). The infraspinatus (ISP) tendon length was measured on the axial TSE sequence, from the myotendinous junction to its insertion on the greater tuberosity of the humerus (Fig. 5). The thickness of the ISP tendon was measured at the insertion of the tendon on the humeral footprint (Fig. 6). Two fully trained radiologists blindly performed the measurements of the length, thickness and width of the SSC, SSP and ISP tendons. Three measurements were taken from each examiner for each parameter considered, and the mathematical average of the 6 performed measures was used for statistical purposes. Measurements were performed by two independent observers specialized in shoulder surgery.

### Statistical analysis

Numerical data were summarized using descriptive statistics as median, range (i.e., minimum and maximum value), mean and standard deviation. The non-normal distribution of the variables was assessed with Shapiro-Wilk test and a non-parametric test (Mann Whitney U test) was used to compare the distribution of values between the two groups (PTRCT and noPTRCT). Analyses were performed with IBM SPSS Statistics for Windows, Version 26.0. Armonk, NY: IBM Corp. P-values < 0.05 were considered statistically significant.

## Results

### Demographic characteristics

Overall, 500 patients were included in the study, 231 females (46.2%) and 269 males (53.8%). The mean age of the patients was 49 ± 12.7 years (range between 20 and 77 years old).

### Measurements of thickness

A statistically significant difference was found between the two groups in the thickness of SSP tendon (p < 0.001). The mean thickness of the SSP tendon was 5.7 ± 0.6 mm (median 6 mm; range 3.9–7.7 mm) in Group 1, 5.9 ± 0.6 mm (median 5.7 mm; range 3.8–7.7 mm) in Group 2. Statistically significant differences were found between the two groups in the thickness of ISP and SSC tendon (p = 0.063 and p = 0.114, respectively) (Table 1).

### Measurements of length

A statistically significant difference was found between the two groups in the length of ISP tendon (p = 0.004). The mean length of the ISP tendon was 27.4 ± 3.2 mm (median 26.6 mm; range 21.9–42.3 mm) in Group 1, 28.3 ± 3.8 mm (median 27.4 mm; range 21.6–42 mm) in Group 2. No statistically significant differences were found between the two groups in the length of SSP and SSC tendon (p = 0.605 and p = 0.173, respectively) (Table 1).

### Measurements of width

Statistically significant differences were found between the two groups in the width of SSP and ISP tendon (p = 0.004 and p = 0.02, respectively). The mean width of the SSP tendon was **17 ±** 1.6 mm (median 16.7 mm; range 13.3–22.1 mm) in Group 1, 17.6 **±** 2 mm (median 17.5 mm; range 12.8–25.7 mm) in Group 2. The mean width of the ISP tendon was 17.7 ± 1.4 mm (median 17.4 mm; range 14.2–21.9 mm) in Group 1, 18.3 ± 2.1 mm (median 17.9 mm; range 14.2–27.1 mm) in Group 2. No statistically significant difference was found between the two groups in the width of SSC tendon (p = 0.749) (Table 1).

**Table 1:** Results of MRI measurements of subjects in Group1 (PTRCT) and Group 2 (no PTRCT), reporting mean, standard deviation, minimum and maximum values and p-value

	Tendon	PTRCT (n = 100) ^$^	noPTRCT (n = 396) ^$^	p-value
Thickness	SSP	5.7 ± 0.6 (3.9–7.7)	5.9 ± 0.6 (3.8–7.7)	< 0.001*
	ISP	5.4 ± 0.5 (3.6–6.5)	5.3 ± 0.6 (3.1–6.8)	0.063
	SSC	6.6 ± 1.2 (3.4–14.7)	6.4 ± 1.0 (3.5-9)	0.114
Length	SSP	24.9 ± 2.3 (13.6–31.4)	25.1 ± 2.3 (19.8–31.4)	0.605
	ISP	27.4 ± 3.2 (21.9–42.3)	28.3 ± 3.8 (21.6–42)	0.004*
	SSC	37.3 ± 4.6 (26.5–47.2)	36.6 ± 4.9 (24.3–49.1)	0.173
Width	SSP	17 ± 1.6 (13.3–22.1)	17.6 ± 2.0 (12.8–25.7)	0.004*
	ISP	17.7 ± 1.4 (14.2–21.9)	18.3 ± 2.1 (14.2–27.1)	0.02*
	SSC	20.1 ± 1.5 (16.3–23.9)	20.3 ± 1.9 (15-31.6)	0.749

$= Median (range, i.e., min - max); Mean ± SD

*= Statistically significant (i.e., p < 0.05)

### Overall measurements

Overall measurements have been grouped so as to assess the differences in tendon sizes among patients positive and negative for PTRCTs. Furthermore, the grouping of these measurements could be of use in further anatomical studies with the objective of assessing rotator cuff tendon footprint.

Overall mean value for RC tendon thickness was 6 ± 1 mm (median 5.7 mm; range: 3.4–14.7 mm) in Group 1 and 5.8 ± 0.9 mm (median 5.9 mm; range: 3.1-9 mm) in Group 2 (p < 0.001) (Table 2). A statistically significant difference was found between the two groups. Therefore, tendons were slightly thicker in patients positive for PTRCTs.

The overall mean length of the RC tendons is 29.8 ± 6.4 mm (median 27.1 mm; range: 13.6–47.2 mm) in Group 1, while it is 30 ± 6.2 mm (median 27.8 mm; range: 19.8–49.1 mm) in Group 2 (p = 0.346) (Table 2). No statistically significant difference was found between the two groups.

The overall mean width for RC tendons is 18.3 ± 2 mm (median 17.9 mm; range: 13.3–23.9 mm) in Group 1 and 18.7 ± 2.3 mm (median 18.4 mm; range: 12.8–31.6 mm) in Group 2 (p = 0.005) (Table 2). A statistically significant difference was found between the two groups. Tendons were reported to be wider in patients negative for PTRCTs.

**Table 2:** Overall results of MRI measurements of subjects in Group1 (PTRCT) and Group 2 (no PTRCT), reporting mean, standard deviation, minimum and maximum values and p-value

	PTRCT (n = 100) ^$^	noPTRCT (n = 396) ^$^	p-value
Thickness	6 ± 1 (3.4–14.7)	5.8 ± 0.9 (3.1-9)	< 0.001*
Length	29.8 ± 6.4 (13.6–47.2)	30 ± 6.2 (19.8–49.1)	0.346
Width	18.3 ± 2 (13.3–23.9)	18.7 ± 2.3 (12.8–31.6)	0.005*

$= Median (range, i.e., min - max); Mean ± SD

*= Statistically significant (i.e., p < 0.05)

Comparison between groups based only on the presence or absence of partial rupture can lead to bias. Therefore, a multivariate analysis to control for confounding factors like age and gender was performed (Table 3). The patients were divided into two groups according to age class: 30–60 and 60–90. Only two items resulted in statistically significant: age 60–90 and SSP thickness. Therefore, it is possible to note that high SSP thickness values in older patients are linked with a higher probability of finding PTRCTs.

**Table 3:** Results of multivariate analysis for age, gender and RC tendon size

Coefficients:	Estimate	Standard deviation	Error z	Value	Pr(>|z|)
(Intercept)	-3,459	3,582	-0,966	0,334	
AGE (30,60]	-0,916	0,509	-1,799	0,072	,
AGE (60,90]	-1,695	0,543	-3,125	0,002	**
SEX M	-0,115	0,275	-0,418	0,676	
SSP_THICKNESS	-0,715	0,251	-2,852	0,004	**
SSP_ LENGTH	0,022	0,064	0,351	0,725	
SSP_ WIDTH	0,093	0,094	0,989	0,323	
ISP_ THICKNESS	0,537	0,324	1,657	0,097	,
ISP_ LENGTH	0,035	0,047	0,748	0,454	
ISP_ WIDTH	0,077	0,089	0,867	0,386	
SSC_ THICKNESS	0,073	0,162	0,452	0,652	
SSC_ WIDTH	0,001	0,033	0,018	0,986	
SSC_ WIDTH	0,085	0,084	1,017	0,309	

SSP: supraspinous tendon; ISP: infraspinous tendon; SSC: subscapularis tendon; Signif. codes: 0 ‘***’ 0.001 ‘**’ 0.01 ‘*’ 0.05 ‘.’ 0.1 ‘ ’ 1, (Dispersion parameter for binomial family taken to be 1) Null deviance: 406.56 on 339 degrees of freedom. Residual deviance: 376.61 on 327 degrees of AIC: 402.61. Number of Fisher Scoring iterations: 4

## Discussion

Various biomechanical studies have collected preliminary data about shoulder function [40, 41], but further insights are still needed regarding RC tendon characteristics. In the present diagnostic study, starting from a 1758 patient sample, the length, thickness and width of the SSC, SSP and ISP tendons in 500 subjects was measured. The mean thickness of RC tendons was less than 6 mm. In PTRCTs group the mean value of thickness of the SSP tendon was 5.7 ± 0.6 mm, while in the control group was 5.9 ± 0.6 mm. Therefore, statistical differences were found between the two groups.

The current trend is to repair PTRCTs involving more than 50% tendon thickness [21]. Therefore, a lesion involving more than 3 mm of the RC should be repaired. However, considering that no differences were reported between two groups, it is not possible to define and validate by MRI a cut-off value to decide for a surgical repair of a partial lesion. Therefore, the mean values obtained should be considered with the purpose of establishing a universal cut-off value, but further clinical and diagnostic studies are needed to refine it.

Current uncertainties in the literature regarding this topic significantly impair the choice of the appropriate therapeutic approach in each different situation. This could be remarkably facilitated by precisely outlining a standard value for RC tendon characteristics. Shoulder models accounting for the length, thickness and width of the different tendons can significantly improve the understanding of the rotator cuff area [42].

Conservative management is usually the first treatment option for PTRCTs [43], given that most patients recover from symptoms within 12 to 18 months. However, the most effective treatment in isolation is yet to be defined and should be tailored to individual patient needs. Several surgical approaches have been proposed to manage these lesions, but the optimal treatment is still debated. Petrillo et al. [44] have deemed reverse shoulder arthroplasty helpful in restoring pain-free range of motion and improving shoulder function in different types of RC tear. Additionally, arthroscopic rotator cuff repair has shown promising clinical results, even considering a long-term follow up [45–49]. The conversion into full-thickness tear and consequent repair in a traditional fashion [50, 51] has been a standard treatment option for decades [52]. Carroll et al. [26] proposed using an intra-articular depth guide in the measurement of PTRCT. In recent times, experimental treatments using stem cells and growth factors [53] have been developed. Although they have demonstrated concrete validity, further research must be conducted to assess possible future implications better. Though promising, most of these techniques do not allow for accurate preoperative evaluation of RC tear thickness and comparison with standard values. This is because exact RC tendon thickness had not yet been evaluated.

However, the thickness, width, and length of RC tendons can be measured using different methods, each with intrinsic limitations. Cadaver studies represent a long-lasting trend in almost all types of medical research. Furthermore, using cadaveric shoulders offers the possibility to observe the tendon and measure it directly. However, anatomy is amended after cadaveric dissection, and the costs of this study type are particularly stringent [54]. Lastly, the fact that measurements are obtained using a caliper, which only considers the largest portion of the tendon, makes this study type rather limited [42]. Significant differences have been found comparing cadaver tendon measurements with those of live subjects. Further studies are needed to expand on this discrepancy.

On the other hand, studies utilizing ultrasound allow the user to visualize tendon thickness in vivo with similar accuracy to MRI, although the latter can bring more detail [35]. However, limitations are not excluded. This type of imaging technique projects three-dimensional images on a two-dimensional plane [42]. Besides this projection error, a more practical limitation is represented by the fact that if the transducer is not perfectly perpendicular to the measured structure, the resulting measurement will be oblique.

A more reliable technique to investigate tendon anatomy is MRI, which is of great utility in RC imaging [20, 55]. Additionally, it may provide information on muscle degeneration and other pathologic processes already present in the analyzed shoulder [36].

This study assesses the exact size of rotator cuff tendons in healthy and PTRCT patients using MRI. Moreover, two fully trained radiologists blindly performed the measurements of RC tendons. According to the present literature, strict exclusion criteria were applied to reduce the sample size [56–58]. This may represent a significant step forward in assessing a general value for RC tendon width, length and thickness. As a diagnostic study, the precise values obtained from a large sample size contribute to the clinical relevance of this paper. Lastly, MRI was the imaging tool of choice for the previously discussed reasons.

### Limitations

Even though MRI represents one of the best methods for conducting this type of research [59–61], it also has limitations. For example, patients may not tolerate or bear contraindications for MRI [62]. Furthermore, metal implants on patients may lead to the presence of severe artifacts on imaging [63]. The lack of intra/inter-observer reliability poses as a limitation as well. Lastly, 1.5T MRI used in the present study has lower diagnostic power as compared to a 3T MRI, but remains more diffused in clinical practice and thus more reproducible.

## Conclusions

The data present in this paper may serve as a tool for surgeons to properly manage PTRCTs. The findings of the present study aimed to provide a better understanding of the anatomy of the rotator cuff, while future studies may be helpful in defining the optimal treatment for PTRCTs. Indeed, MRI is of great utility in detecting partial and full-thickness rotator cuff tears, allowing the surgeon to properly assess the size of the tear and to select the most appropriate mode of treatment. Additionally, an increase in diagnostic accuracy could improve both conservative and surgical approaches. Lastly, further clinical trials using more accurate diagnostic MRI tools are required to better define the anatomical differences between PTRCT and healthy patients.

## Data Availability

The datasets used and/or analysed during the current study are available from the corresponding author on reasonable request.
